# Prediction of trapezius muscle activity and shoulder, head, neck, and torso postures during computer use: results of a field study

**DOI:** 10.1186/1471-2474-15-292

**Published:** 2014-09-03

**Authors:** Jennifer L Bruno Garza, Belinda HW Eijckelhof, Maaike A Huysmans, Peter W Johnson, Jaap H van Dieen, Paul J Catalano, Jeffrey N Katz, Allard J van der Beek, Jack T Dennerlein

**Affiliations:** Department of Environmental Health, Harvard University, Boston, USA; Division of Occupational and Environmental Medicine, UConn Health, Farmington, USA; Department of Public and Occupational Health VU University Medical Center, Amsterdam, The Netherlands; EMGO Institute for Health and Care Research, VU University Medical Center, Amsterdam, The Netherlands; Body@Work Research Center on Physical Activity, Work and Health, TNO-VU/VUmc, Amsterdam, The Netherlands; Department of Environmental Health, University of Washington Seattle, Seattle, USA; Faculty of Human Movement Sciences, VU University, Amsterdam, The Netherlands; Department of Biostatistics, Harvard School of Public Health Boston, Boston, USA; Dana Farber Cancer Institute Boston, Boston, USA; Department of Epidemiology, Harvard School of Public Health, Boston, USA; Division of Rheumatology, Immunology and Allergy, Brigham and Women’s Hospital, Boston, USA; Department of Orthopaedic Surgery, Brigham and Women’s Hospital, Boston, USA; Department of Physical Therapy, Bouvé College of Health Sciences, Northeastern University, Boston, USA

## Abstract

**Background:**

Due to difficulties in performing direct measurements as an exposure assessment technique, evidence supporting an association between physical exposures such as neck and shoulder muscle activities and postures and musculoskeletal disorders during computer use is limited. Alternative exposure assessment techniques are needed.

**Methods:**

We predicted the median and range of amplitude (90^th^-10^th^ percentiles) of trapezius muscle activity and the median and range of motion (90^th^-10^th^ percentiles) of shoulder, head, neck, and torso postures based on two sets of parameters: the distribution of keyboard/mouse/idle activities only (“task-based” predictions), and a comprehensive set of task, questionnaire, workstation, and anthropometric parameters (“expanded model” predictions). We compared the task-based and expanded model predictions based on R^2^ values, root mean squared (RMS) errors, and relative RMS errors calculated compared to direct measurements.

**Results:**

The expanded model predictions of the median and range of amplitude of trapezius muscle activity had consistently better R^2^ values (range 0.40-0.55 compared to 0.00-0.06), RMS errors (range 2-3%MVC compared to 3-4%MVC), and relative RMS errors (range 10-14%MVC compared to 16-19%MVC) than the task-based predictions. The expanded model predictions of the median and range of amplitude of postures also had consistently better R^2^ values (range 0.22-0.58 compared to 0.00-0.35), RMS errors (range 2–14 degrees compared to 3–22 degrees), and relative RMS errors (range 9–21 degrees compared to 13–42 degrees) than the task-based predictions.

**Conclusions:**

The variation in physical exposures across users performing the same task is large, especially in comparison to the variation across tasks. Thus, expanded model predictions of physical exposures during computer use should be used rather than task-based predictions to improve exposure assessment for future epidemiological studies. Clinically, this finding also indicates that computer users will have differences in their physical exposures even when performing the same tasks.

**Electronic supplementary material:**

The online version of this article (doi:10.1186/1471-2474-15-292) contains supplementary material, which is available to authorized users.

## Background

Because of the high incidence and prevalence of musculoskeletal disorders and their symptoms that has been noted among computer users, computer use is believed to be an important determinant of musculoskeletal disorders and their symptoms [1–4]. Increased exposure to physical factors such as higher and less variable muscle activity or non-neutral and less variable postures during computer work may explain this association [5–8]. Yet, evidence from epidemiological studies supporting this pathway is limited, largely due to the difficulties associated with measuring these physical exposures reliably and accurately enough in large enough samples of workers performing their own computer work [9]. To date, most epidemiological studies that have examined physical exposures during computer use have been limited to one-time, single observed measurements. For example, Marcus et al. [10] used a single measurement of posture to represent each worker’s exposure during computer use, and found few associations with musculoskeletal symptoms. Other metrics of these physical exposures such as the intensity and variability over a work period, which require measurements taken directly and continuously over a work period, are believed to be associated with musculoskeletal symptoms if this relationship could be investigated [5, 11].

As an alternative to direct and continuous measurements, several studies have predicted occupational physical exposures using statistical models developed from smaller cohort studies where exposures were measured directly along with parameters such as task, workstation characteristics, and individual factors. Task, especially, has been used to predict certain physical exposures accurately in situations where there is large variation in these exposures across tasks and small variation in exposures within tasks. For example, Li et al. [12] accurately estimated factory workers’ exposures to noise, and Methner et al. [13] estimated factory workers’ exposures to nanoparticles, using task-based models. When there is large variation in exposures within individuals performing the same task, task is not sufficient to explain exposures, and other parameters can be used to obtain accurate predictions of exposures. Chen et al. [14] predicted vibration dose for taxi drivers based on variables such as driving speed, car type, and engine size. Using these predictions, the authors were able to report an association between vibration exposure and low back pain among taxi drivers. Van der Beek et al. predicted exposure to lifting based on the amount and type of scaffolding assembled by workers [15–17]. In the field of computer work, Bruno Garza et al. [18] used both task and anthropometric parameters to predict muscle activities and postures during computer use, with limited success. Their predictions of muscle activities, especially, corresponded to very low R^2^ values and high RMS and relative RMS errors. However, this study was based on data collected in the laboratory with a limited number of parameters measured.

Therefore, the goal of this study was to evaluate predictions of the median and range of amplitude of trapezius muscle activity and the median and range of motion of shoulder, head, neck, and torso postures based on measurements collected during computer use in a real-life work setting. We compared two methods for predicting muscle activities and postures, based on only the distribution of keyboard/mouse/idle tasks (task-based predictions) or on a comprehensive set of 104 task, questionnaire, workstation, and anthropometric parameters (expanded model predictions).

## Methods

### Study design and participant recruitment

Data used for this study included computer interactions, questionnaire responses, measurements of workstation setup and anthropometry, and continuous measurements of muscle activity of the right and left trapezius, and shoulder, head, neck, and torso posture data from 117 office workers (33 male, 84 female) performing computer work while working for approximately two hours at their own workstations. A two hour time period has been shown to capture representative measurements of workday physical exposures during computer use [19, 20]. Workers were recruited from 9 departments at the VU University and VU University Medical Center in Amsterdam, the Netherlands [21–23]. Eligible workers worked more than 20 hours per week, were free of musculoskeletal pain the week prior to measurement, were used to working with the mouse with the right hand, and could use a desktop computer during the measurement period. All workers described themselves as “office workers”, and performed a variety of typical computer tasks during the measurement period such as word processing, internet research, emailing, etc. The measurements were balanced so that half were taken in the morning and the rest in the afternoon. This project was approved by the applicable Institutional Review Boards for protection of human subjects (Harvard School of Public Health Office of Regulatory Affairs and Research Compliance, protocol #17938-105, The Medical Ethics Committee Independent Review Board of the VU University Medical Center in Amsterdam, registered with the US Office of Human Research Protections as protocol #IRB0002991). Informed consent for participation in the study was obtained from participants, who were all adults.

### Trapezius muscle activity and shoulder, head, neck, and torso postures measurements

Muscle activity of the right and left upper trapezius was measured using the Mega WBA wireless logger system (Mega Electronics LTD, Kupio, Finland). Electrodes were mounted in accordance with published guidelines for the surface EMG of the trapezius while participants were sitting in the posture that would be assumed during computer use [24]. Data were recorded at 1000 samples per second after amplification (bandwidth of 10–500 Hz), were rectified and smoothed through a 3 Hz second-order, zero phase, low-pass Butterworth filter, and were down-sampled to 40 samples per second using a mean filtering procedure. All data were normalized to each participant’s highest 1-second average maximum voluntary contraction (MVC). Three MVCs with approximately 1 minute of rest in between were collected while participants attempted to elevate their shoulders upwards against resistance applied by the experimenter.

Shoulder abduction and flexion and head, neck, and torso flexion and lateral tilt were measured using five G-Link Data Loggers (Microstrain, Inc; Williston, VT) containing triaxial accelerometers. The accelerometers have a range from -/+2 g (gravitational acceleration) with an accuracy of 10 mg and resolution of 1.5 mg. Inclinometers are a reliable and accurate method for measuring upper extremity postures [25, 26]. Sensors were mounted on each arm as close to the shoulder joint as possible and centered on the forehead using stretchable bands, and on the torso centered below the acromial notch and on the neck centered above the C7 vertebrae using tape. Data were logged at 25 samples per second, downloaded to a personal computer, and filtered using a 5 Hz second order, zero-phase, low-pass Butterworth filter. Before being converted from acceleration units to degrees, the accelerometer data were transformed from the sensor’s coordinate system to the anatomic coordinate system defined by a reference posture (standing erect looking straight ahead with arms resting at sides), and were aligned with flexion and extension (bowing forward at the hips only).

Shoulder rotation was measured using a validated, single video camera-based system that calculated angles based on the projected position on black and white markers taped at the dorsal side of the wrist, the lower biceps brachii (on the distal part of the upper arm, above the elbow crease), and the acromion [27]. Video images were collected at 30 frames per second, downloaded to a personal computer, and converted to position data before being filtered using a 5 Hz fourth-order, low-pass filter.

All measured trapezius muscle activity and shoulder, head, neck, and torso postures were separated into the median and range of amplitude (for trapezius muscle activity) or range of motion (for postures) during all computer activity, keyboard activity only, mouse activity only, and idle activity only as described in [22]. Range of amplitude of trapezius muscle activity and range of motion of posture were defined as the difference between the 90^th^ percentile and 10^th^ percentile values. The mean, minimum, and maximum values of all median and range of amplitude of trapezius muscle activities and median and range of motion of shoulder, head, neck, and torso postures across all participants are summarized in Table 1.

**Table 1:** **Mean, minimum, and maximum values of the median and range of amplitude of trapezius muscle activity and the median and range of motion of shoulder, head, neck, and torso postures across all participants (n = 117)**

	Median			Range of Amplitude/Range of Motion		
	Mean	Minimum	Maximum	Mean	Minimum	Maximum
*Shoulder EMG (%MVC)*						
Right Trapezius	5.43	0.33	16.19	7.90	0.82	28.61
Left Trapezius	4.29	0.29	18.30	7.91	0.82	28.61
*Shoulder Posture (degrees)*						
Right Abduction	13.12	-34.08	39.96	15.30	1.67	62.15
Left Abduction	7.17	-29.66	29.31	16.56	5.13	53.20
Right Flexion	12.08	-23.50	46.53	24.42	2.71	52.31
Left Flexion	12.71	-14.62	41.19	26.52	7.35	56.15
Right Internal Rotation	-2.33	-28.87	49.83	44.47	10.47	77.64
Left Internal Rotation	22.31	-18.56	60.63	28.52	5.60	71.32
*Head Posture (degrees)*						
Tilt	-0.94	-20.54	9.27	11.04	4.37	80.70
Flexion	12.14	-13.76	43.31	26.94	4.01	67.29
*Neck Posture (degrees)*						
Tilt	-0.88	-18.43	8.39	8.47	3.35	21.80
Flexion	11.49	-34.96	31.64	15.63	4.34	47.09
*Torso Posture (degrees)*						
Tilt	-1.90	-19.54	4.43	7.56	2.69	38.29
Flexion	13.89	-15.64	50.07	17.67	4.12	43.58

### Computer interaction monitor (Measurement of tasks)

Duration of computer, keyboard, mouse, and idle activities defined our tasks and were identified using either computer interaction monitoring (CIM) software that was installed onto each participant’s computer or run through an external USB tracker (Model 110b, Ellisys Inc., Geneva, Switzerland) during the measurement and aligned with the muscle activity and posture data using a series of distinctive movements that would show in the data streams of multiple systems. Keyboard and mouse activities were defined as any series of keyboard events (keystrikes) or mouse events (mouse movement, scrolling, or button clicks), respectively, with less than 2 seconds of inactivity between. Idle activities were defined as any time there were no keyboard or mouse activities for at least two seconds but less than 30 seconds. The combination of keyboard activities, mouse activities, and idle activities together were considered periods of computer activities, for which the summary statistics for the muscle activities and postures were calculated. Data from non-computer activities (no keyboard, mouse or idle activities for at least 30 seconds) were not included in the following analyses. The percentage of keyboard time, percentage of mouse time, and percentage of idle time were calculated as the duration of keyboard, mouse, and idle activities, respectively, divided by the total computer activity duration, and these were used as our task variables.

### Questionnaire, workstation, and anthropometry measurements

On the day of their measurements, participants completed a questionnaire from the PROMO study, a two-year prospective study of office workers [28–30]. The questionnaire contained items concerning individual demographics, self-reported computer workstation set up [31], psychosocial scales [32–37], musculoskeletal complaints, job characteristics, and leisure time activities. In addition, the experimenter measured participant’s anthropometry and workstation set up using methods described in Won et al. and Marcus et al. [10, 38]. The 104 parameters chosen for this study represented all parameters that we expected based on previous epidemiologic studies and theorized relationships could be related to our physical exposures during computer use [10, 21, 22, 31, 38]. A complete list of the parameter categorization and references can be found in Appendix A.

### Task-based predictions

The task-based predictions were calculated as the time weighted average of the median and range of amplitude values of the muscle activity and the median and range of motion of postures during keyboard, mouse, and idle activities averaged across participants and reported in [21]. The weights for the time weighted averages were the percentage of keyboard time, percentage of mouse time, and percentage of idle time measured by the computer interaction monitor for each participant. Hence, task-based estimates were calculated based on the assumption that there is large variation in muscle activities and postures across tasks and small variation in exposures within tasks.

### Expanded model predictions

The expanded model predictions were calculated from general linear models incorporating parameters associated with each median and range of amplitude trapezius muscle activity or median and range of motion shoulder, head, neck, or torso posture (proc glm, SAS v.9.2 SAS Institute Inc., Cary, NC USA). Parameters used to generate predictions were selected via a four-step procedure. *Step 1*: we identified all parameters that, via univariate analysis of variance (ANOVA) analyses (proc glm, SAS v.9.2 SAS Institute Inc., Cary, NC USA), were associated with each muscle activity or posture (two sided p < 0.20) *Step 2*: since there was a large number of parameters associated with each physical exposure, we categorized the 104 parameters into seven categories (individual characteristics, job characteristics, computer work behaviors, psychosocial factors, workstation setups, health outcomes, and leisure time parameters) as shown in Appendix A. *Step 3*: by group, including all parameters identified in Step 1, we employed a backwards selection procedure (proc glmselect) using a significance criterion with p < 0.20. A liberal p-value of 0.20 was chosen to allow more parameters into the first stages of the selection process. *Step 4*: including the parameters from all of the seven groups identified in the Step 3, we selected the final parameters via a backwards selection procedure using a significance criterion of p < 0.10. The p-value of p < 0.10 was chosen as the selection criteria because it minimized the Akaike Information Criterion (AIC) and the Schwarz Bayesian Criterion (SBC) (measures of the relative quality of the model based on goodness of fit and model complexity) and maximized the adjusted R^2^ values when comparing p < 0.05, p < 0.10, and p < 0.20 cutoffs, thus allowing for maximal model performance while simultaneously reducing the likelihood of overfitting the models or introducing parameters into the models by chance [39]. Visual inspection for normality of the residuals for each model confirmed that parametric methods were appropriate.

### Evaluation of task-based and expanded model predictions

Three values were calculated to ascertain the quality of our predictions. R^2^ values were calculated by performing a simple linear regression of the predicted and measured median, range of amplitude, and range of motion values. R^2^ values describe the percent variability in the observed values explained by the predicted values. Root mean squared (RMS) errors were calculated by subtracting the predicted values from the corresponding observed values, squaring them, and then taking the square root of the averaged squared errors. RMS errors describe the averaged absolute differences between observed and predicted values (average residuals). Relative RMS errors were determined by dividing the RMS error by the full range of median, range of amplitude, or range of motion values observed (maximum-minimum values shown in Table 1). Relative RMS errors describe the average residuals while taking into account the variation in the observed values (normalized residuals). Because there can be bias in estimating the performance of regression models that were built using the same measurements that we are predicting, the R^2^, RMS, and relative RMS values for the expanded models were calculated as averages given by the jackknife method [14, 40]. That is, we calculated 117 R^2^, RMS, and relative RMS values, one set of values for each model after dropping all measurements from one participant, and then calculated the average values across all 117 iterations.

We calculated the percent increase in participants that would be required in order for our physical exposure predictions to have the same power to detect differences as the direct measurements. We assumed that this percent increase is due to the increased standard deviation of the predicted values as a result of reduced statistical precision. Thus, we assumed that there is no systematic misclassification bias. The percent increase in participants was calculated as the square root of R^2^ divided by the slope of the simple linear regression used to calculate R^2^.

## Results

The expanded model predictions of median trapezius muscle activities and shoulder, head, neck, and torso postures were positively correlated with the measured values, with R^2^ values ranging from 0.22 to 0.58. Except for shoulder internal rotation, which had an R^2^ value of 0.35, R^2^ values for the task-based predictions were an order of magnitude less than for the comprehensive predictions, ranging from 0.01 to 0.10. RMS errors for right and left median trapezius muscle activity were each 3%MVC for the task-based predictions, range from 2-3%MVC for the expanded model predictions. RMS errors for postures ranged from 13 to 22 degrees for the task-based predictions, and ranged from 3 to 14 degrees for the expanded model predictions. Relative RMS errors ranged from 13 to 22% for the task-based predictions and from 11 to 19% for the expanded model predictions (Table 2). There was a large range of parameters included in the expanded model predictions across the seven categories (Table 3). Four parameters (#45 “sitting posture”, #64 “today’s stress”, #78 “key displacement”, and #93 “acquaintances with symptoms”) were used to predict four to five physical exposures each. Either the “percentage keyboard use” (#46) or “percentage mouse use” (#47), or “percentage idle time” (#48) task parameters were used to predict six of the fourteen physical exposures.

**Table 2:** **Predictions of median trapezius muscle activity and shoulder, head, neck, and torso postures**

Median	Task-based predictions			Expanded model predictions^1^		
	*R* ^*2*^	RMS	Relative RMS	*R* ^*2*^	RMS	Relative RMS
*Shoulder EMG (%MVC)*						
Right Trapezius	0.06	3	19	0.48	2	14
Left Trapezius	0.03	3	18	0.55	3	14
*Shoulder Posture (degrees)*						
Right Abduction	0.01	10	14	0.58	7	10
Left Abduction	0.03	9	15	0.35	7	12
Right Flexion	0.01	14	20	0.22	13	18
Left Flexion	0.02	13	22	0.39	10	19
Right Internal Rotation	0.35	15	19	0.48	14	17
Left Internal Rotation	0.09	12	15	0.35	11	14
*Head Posture (degrees)*						
Tilt	0.00	4	13	0.24	4	13
Flexion	0.02	9	16	0.32	8	14
*Neck Posture (degrees)*						
Tilt	0.00	3	13	0.36	3	11
Flexion	0.10	9	13	0.47	7	11
*Torso Posture (degrees)*						
Tilt	0.00	3	14	0.44	3	11
Flexion	0.00	11	16	0.33	9	14

^1^R^2^, RMS, and Relative RMS are the average values from jackknifed calculations.

Comparison of task-based and expanded model predictions based on R^2^ values, RMS errors, relative RMS errors.

**Table 3:** **Parameters in each category used in the expanded model predictions of median trapezius muscle activity and shoulder, head, neck, and torso posture**

Median	Individual factors (Total = 16)	Job characteristics (Total = 12)	Computer work behaviors (Total = 20)	Psychosocial factors (Total = 17)	Workstation setup (Total = 27)	Health and pain (Total = 6)	Leisure time activities (Total = 6)	Key (p-values)	Mouse (p-values)	Idle (p-values)	Total number of predictors
*Shoulder EMG (%MVC)*											
Right Trapezius^1^	16		30,37,41,46	56,64	71,87	93		<0.01			10
Left Trapezius	2,10	18,26	29,37	60,64	76,78	93	102				12
*Shoulder Posture (degrees)*											
Right Abduction	2		40	52,61,65	76,77,82,85	93					10
Left Abduction	4,11		33,38,43		72,82		102				8
Right Flexion				51,60,65			102				4
Left Flexion	2		45	67,81,85		93					6
Right Internal Rotation	16	20,22	33,45,46,47	53,58,55,63				0.05	< 0.01		11
Left Internal Rotation			30,45,46,48	52,64,65	84,92			<0.01		0.05	9
*Head Posture (degrees)*											
Tilt		22	43	59,61	78	98					6
Flexion		24	47	52	77,78,83,88,89	96			0.01		9
*Neck Posture (degrees)*											
Tilt		17	42,43	70,72,78,85							7
Flexion		17,21	29	55,59	67,72,81,89,90						10
*Torso Posture (degrees)*											
Tilt		24	29,35	64	66,78	98					7
Flexion			45	55,59	74,78,89						6

Numbers in each category correspond to the numbers assigned to each parameter described in Appendix A.

Expanded model predictions of the range of amplitude of trapezius muscle activity and the range of motion of shoulder, head, neck, and torso postures also had much larger R^2^ values and smaller RMS and relative RMS errors than the task-based predictions (Table 4). R^2^ values ranged from 0.22 to 0.43 for the expanded model predictions, and from 0.00 to 0.05 for the task-based predictions. RMS errors for range of amplitude of right and left trapezius muscle activity were each 4%MVC for the task-based predictions, and ranged from 3 to 4%MVC for the expanded model predictions. RMS errors for postures ranged from 3 to 22 degrees for the task-based predictions, and ranged from 3 to 14 degrees for the expanded model predictions. Relative RMS errors ranged from 14 to 42% for the task-based predictions and from 9 to 21% for the expanded model predictions. There was a large range of parameters included in the expanded model predictions across the seven categories (Table 5). Eight parameters (#4 “education level”, #29 “use of more than one computer at the same time during computer use”, #41 “able to touch type”, #62 “stress at work”, #69 “monitor location”, #72 “chair height”, #75 “mouse type”, and #104 “leisure computer use”) were used to predict four to six physical exposures each. The “percentage keyboard use” (#46) or “percentage mouse use” (#47), task parameters were used to predict three of the fourteen physical exposures.

**Table 4:** **Predictions of range of amplitude of trapezius muscle activity and range of motion shoulder, head, neck, and torso postures**

Range of Amplitude/Range of Motion	Task-based predictions			Expanded model predictions^1^		
	R^2^	RMS	Relative RMS	R^2^	RMS	Relative RMS
*Shoulder EMG (%MVC)*						
Right Trapezius	0.00	4	18	0.40	3	10
Left Trapezius	0.00	4	16	0.43	4	13
*Shoulder Posture (degrees)*						
Right Abduction	0.02	9	16	0.36	8	13
Left Abduction	0.02	8	17	0.35	7	15
Right Flexion	0.02	8	16	0.39	7	14
Left Flexion	0.01	10	19	0.41	8	16
Right Internal Rotation	0.00	21	30	0.22	14	20
Left Internal Rotation	0.04	15	23	0.36	14	21
*Head Posture (degrees)*						
Tilt	0.00	10	14	0.41	*7*	9
Flexion	0.03	22	35	0.39	9	14
*Neck Posture (degrees)*						
Tilt	0.01	3	16	0.33	3	14
Flexion	0.05	7	16	0.42	*6*	13
*Torso Posture (degrees)*						
Tilt	0.01	16	42	0.34	4	10
Flexion	0.02	11	29	0.30	8	20

^1^R^2^, RMS, and Relative RMS are the average values from jackknifed calculations.

Comparison of task-based and expanded model predictions based on R^2^ values, RMS errors, relative RMS errors.

**Table 5:** **Parameters in each category used in the expanded model predictions of range of amplitude of trapezius muscle activity and range of motion of shoulder, head, neck, and torso postures**

Range of Amplitude/Range of Motion	Individual factors (Total = 15)	Job characteristics (Total = 12)	Computer work behaviors (Total = 20)	Psychosocial factors (Total = 17)	Workstation setup (Total = 27)	Health and pain (Total = 6)	Leisure time activities (Total = 6)	Key (p-values)	Mouse (p-values)	Idle (p-values)	Total number of predictors
*Shoulder EMG (%MVC)*											
Right Trapezius^1^	10,16	17,25	41	53,59	72						8
Left Trapezius	11	17	29,43	64	69,72,78	98					9
*Shoulder Posture (degrees)*											
Right Abduction	4,12	18	44		69,72,77,87	97					9
Left Abduction	2,9,10,11				69,75,82	93	102				9
Right Flexion	4	21	32	51,62,65	80	93	99,103				10
Left Flexion			29,32,41,43	50,51	69	93	104				9
Right Internal Rotation		24	35	58	72,82,89	95					7
Left Internal Rotation	16	24	41,46	53	72,75,86		103	0.03			9
*Head Posture (degrees)*											
Tilt		19,21,28	47	51,62	83	98	102,104		0.03		10
Flexion	4	18	29,36,41	51	83,92						8
*Neck Posture (degrees)*											
Tilt	4	23,26			74,83		101				6
Flexion	4		29,31	62	70,75	97	100,101,104				10
Torso Posture (degrees)											
Tilt	10	24,28	46		70,74,78		102,104	0.03			9
Flexion	3,4,9	18		62	75						6

Numbers in each category correspond to the numbers assigned to each parameter described in Appendix A.

**Figure 1:**
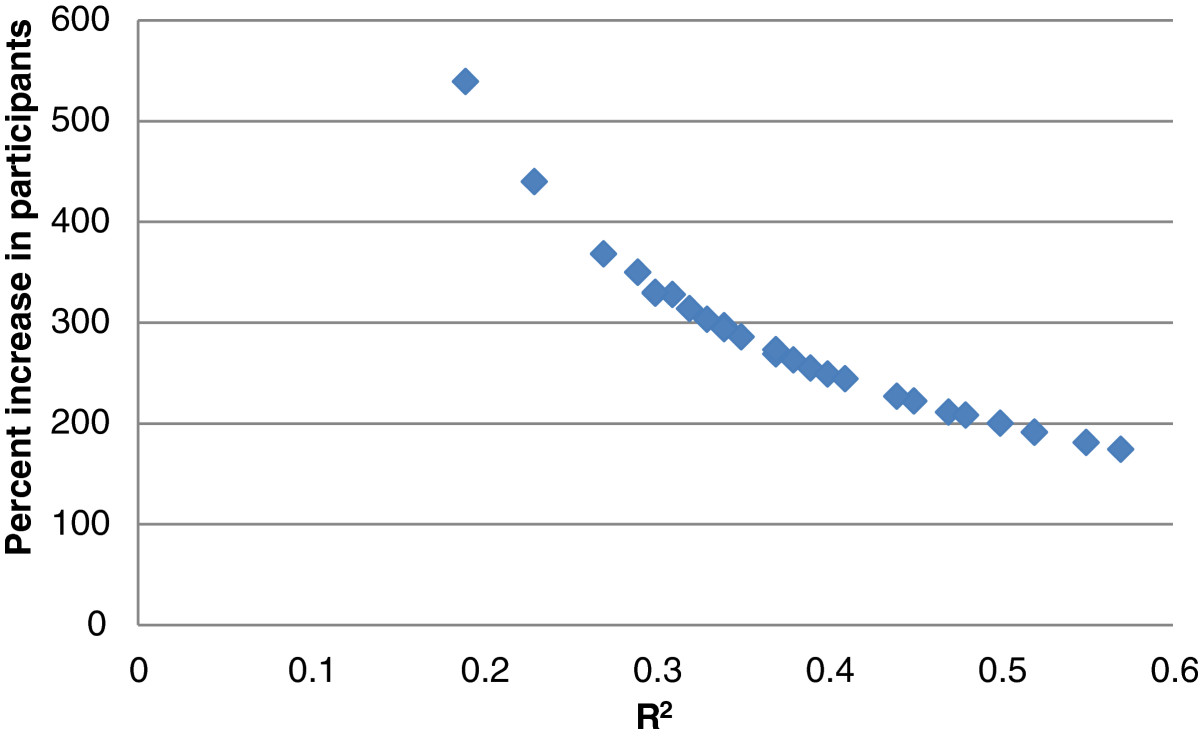
**Percent increase in participants that would be required in order for the expanded model predictions to have the same power to detect differences as the direct measurements, as a function of R** ^**2**^ **, for each median and range of amplitude of trapezius muscle activity and each median and range of motion of shoulder, head, neck, and torso posture.**

The percent increase in participants required for each median and range of amplitude of trapezius muscle activity and each median and range of amplitude of shoulder, head, neck, and torso postures in order to have the same power to detect differences as the direct measurements is inversely proportional to the R^2^ value for that exposure. Since the R^2^ values were consistently larger for the expanded model predictions, a smaller percent increase is needed for the expanded model predictions than for the task-based predictions. The percent increases for each of the median, range of amplitude, and range of motion expanded model predictions are shown in Figure 1.

## Discussion

The goal of this study was to evaluate predictions of the median and range of amplitude of trapezius muscle activity and the median and range of motion of shoulder, head, neck, and torso postures based on measurements collected during computer use in a real-life work setting. The expanded model predictions (based on a comprehensive set of task, questionnaire, workstation, and anthropometric parameters) out-performed task-based predictions (based on the distribution of keyboard/mouse/idle activities only), with much larger R^2^ values, RMS errors, and relative RMS errors, for every median and range of amplitude trapezius muscle activity and median and range of motion shoulder, head, neck, and torso posture. Additionally, only 9 of the 28 full models incorporated task parameters. Based on these results, we can conclude that the variation in muscle activities and postures across users performing the same computer task is large, especially in comparison to the variation across tasks. Methodologically, this finding supports the use of full models including a wide range of task, questionnaire, workstation, and anthropometric parameters to predict physical exposures during computer use instead of the task-based predictions to account for this variation. Clinically, this finding supports the idea that computer users will have differences in their muscle activity and postural exposures even when performing the same tasks.

Our findings that task-based methods do not generate accurate predictions of actual exposures confirm the results of previous studies. Mathiassen et al. [41] reported that task-based exposure predictions were not better than occupation based predictions of trapezius muscle activity for office workers. Similarly, Svendsen et al. [25] reported low correlations between predictions and direct measures of shoulder postures among painters, machinists, and mechanics.

The similarities and differences in the findings of this study compared to a previous laboratory study can inform future prediction methods [18]. Bruno Garza et al. [21] reported low R^*2*^ values and large RMS errors for trapezius muscle activity using predictions based only on task and four variables describing individual factors: age, gender, body mass index, and shoulder width. In the current field study, much better predictions of trapezius muscle activity were produced using mostly parameters from categories other than individual factors. While the quality of predictions of shoulder postures was not very different between the laboratory study and the current study, in this study we demonstrated that these physical exposures can still be predicted as well in the field, where there are likely more sources of variability to affect physical exposures, as in the laboratory [42]. Finally, the current study provided predictions of head, neck, and torso postures, which were not calculated in the laboratory study.

Understanding the unexplained sources of variation within our data sets could lead to improved predictions in future studies. With R^2^ values that ranged from 0.22 to 0.58, we were still unable to capture between 42% and 78% of the variation in our exposures. In this study, we strove to include all parameters that we expected based on previous epidemiologic studies and theorized relationships, could be related to our physical exposures during computer use (e.g. [10, 21, 22, 31, 38]). It is possible that there may be other parameters than the 104 described here that we or others have not yet considered or explored. Another possible reason for this unexplained variation could be that the computer workstation environment affords different strategies for interacting with the computer, allowing participants to perform the same tasks using different postural strategies in a way that can be difficult to capture using measureable variables [43]. Additionally, the workers included in our study population all had fairly well-adjusted workstations, reducing the variation in our dataset and thus making it more difficult to predict this variation. This phenomena has been observed in previous studies of other occupations as well [25].

This study provided some information on the associations between specific parameters and muscle activities and postures. We reported that “use of more than one computer at the same time during computer use” was a used to predict range of amplitude of left trapezius muscle activity as well as range of motion of left shoulder flexion and head and neck flexion postures. There are only a few previous studies which have investigated associations between any of the parameters considered in this study and muscle activities and postures during computer use, and no previous study has demonstrated the association between range of amplitude/range of motion and the use of multiple computers simultaneously. However, this association seems plausible, since it is likely that a computer worker who uses multiple computers would have to move around more within the workstation, increasing the ranges of amplitude and motion. Other variables included in our models have been reported in previous studies to not have an effect on exposures. For instance, Gerr et al. [44] reported that adjustability of workstation chairs did not change neck or upper limb postures, while we used chair height to predict median left shoulder abduction and neck flexion. It is important to consider that we investigated a large group of different parameters which theoretically could have associations with different exposures, but many of these associations had not yet been tested in practice and it was not the primary aim of the study to describe associations between specific parameters and exposures. Our study provides information on potential predictors of different median and range of amplitude of muscle activities and median and range of motion of postures that could be investigated in future work.

We were able to improve predictions substantially when incorporating parameters other than task into the models; however, whether the predictions reported here are good enough to be used for exposure assessment remains to be determined. There are currently no standards to evaluate the occupational predictions. Svendsen et al. [25] concluded that their task-based predictions, which had R^*2*^ correlations mostly below 0.2, were “inefficient” for use in epidemiological studies. Our expanded model R^2^ values, which range from 0.22 to 0.58, are higher than these but lower than the 0.77 to 0.92 reported by van der Beek et al. [17]. In terms of error, Chen et al. [14] concluded that their prediction of whole body vibration, which had a mean relative RMS error of 11%, could be a “useful” method of exposure assessment. Similarly, Xu et al. [45] reported RMS errors of 8-12% for their predictions, and suggested that these models may be “practical” for use in field studies. Our expanded model predictions had relative RMS errors within this range (9-21%). An important test of our predictions, which we were unable to perform in the current study but should be explored in the future, is whether they are able to predict health outcomes.All parameters used for muscle activity and posture expanded model predictions were measured using a questionnaire, computer monitoring software, or tape measure, methods which are significantly less time and cost intensive than using direct measurements. For this reason, even though a larger sample size of workers would be required due to the decreased statistical precision of predictions compared to direct measurements (Figure 1), we believe that these predictions may be useful for examining neck and upper limb muscle activities and postures in future epidemiological studies of physical exposures and musculoskeletal disorders.

We chose to study both the median and range of trapezius muscle activity and shoulder, head, neck, and torso postures because we believe that each of these exposures may be relevant to the development of musculoskeletal symptoms. All muscle activities and postures selected for this study have been identified as potential risk factors for musculoskeletal symptoms during computer use in previous studies (e.g. [10, 31, 46]). Both increases in median and reduction in ranges of muscle activities and postures may cause damage to musculoskeletal tissues [5, 11, 47]. Other exposures and exposure metrics, such as duration or frequency of exposure, may also be related to the development of musculoskeletal symptoms [48]. The viability of predictions of these for exposure assessment could be considered in future studies. However, we should note that duration can already easily be measured with computer interaction monitoring software that is commercially available and that has been used in recent epidemiological studies [29, 49].

The results of this study must be taken with consideration to the study’s limitations. Any interpretation of the individual parameters used to calculate the expanded model predictions must be done with caution. Previous studies have shown that questionnaire responses can be biased, possibly due to overestimation, self-bias selection, health concerns, etc., leading to misclassification of our parameters [31]. We had limited variability within the categories of some of our parameters (as demonstrated by the small standard deviations in Appendix A), which may have limited the utility of these parameters for predicting our physical exposures. The parameters included in the expanded models may also be confounded by each other, or may be correlated with one another. Additionally, our parameter selection procedure required multiple testing. Thus it is possible that some of the parameters identified in our expanded models were included by chance and may not be replicated in other samples. However, all parameters included in this study were chosen because we expected based on previous epidemiologic studies and theorized relationships that they could be related to our physical exposures of interest during computer use (e.g. [10, 21, 22, 31, 38]). Further, the aim of the study was not to identify causal relations between specific parameters and either muscle activity or postures. The expanded models were derived and their performances tested on the same population, which can lead to overfitting as well as bias in estimating model performance. We tried to reduce and characterize this problem by limiting the number of parameters allowed into the expanded models by using the AIC and SBC criteria and by presenting our R^2^, RMS, and relative RMS errors as averages given by the jackknife method. At this stage, no further data were available for validation of our model.

## Conclusions

In conclusion, the results of this study indicate that the expanded model predictions, based on task, questionnaire, workstation, and anthropometric parameters, perform better than the task-based predictions. The method described here may have implications as an alternative exposure assessment technique for future epidemiological studies. Additionally, our findings support the idea that computer users will have differences in their muscle activity and postural exposures even when performing the same tasks.

## Appendix A

Questionnaire, workstation, and anthropometric parameters included in the study. “Measured” indicates that the parameter was measured directly by a researcher. Otherwise, all other parameters were self-reported by participants based on their responses to the questionnaire.

**Individual factors**

1. Age (mean=40 years, standard deviation=11.6 years)
2. Gender (male=28%/female=72%)
3. Handedness (right=87%/left=13%)
4. Education level (none or primary only=2%/lower vocational only=0%/secondary or vocational only=4%/secondary=8%/higher education=86%)
5. Number of years working for current company (mean=8.5 years, standard deviation=8.4 years)
6. Number of years of daily computer use at work (shorter than 1 year=8%/1-2 years=11%/2-5 years=20%/5-10 years=20%/>10 years=41%)
7. Coping (*DeVries et al. 1995, 14 question scale, range 14–56,* mean=35, standard deviation=5)
8. Over-commitment (*Siegrist et al. 2004, 11 question scale, range 0–18*, mean=7, standard deviation=3)
9. Height (mean=175 cm, standard deviation=12.3 cm)
10. Measured weight (mean=73 kg, standard deviation=14.7)
11. Calculated body mass index (mean=24 kg/m^2^, standard deviation=7.4 kg/m^2^)
12. Measured arm length, acromion to radiale (mean=56 cm, standard deviation=5.6 cm)
13. Measured forearm length, radiale to stylion (mean=25 cm, standard deviation=2.1 cm)
14. Measured hand length, distal wrist crease to dactylion (mean=19 cm, standard deviation=13.9 cm)
15. Measured hand breadth, between metacarpale II and V (mean=8.0 cm, standard deviation=2.9 cm)
16. Measured shoulder breadth, acromion to acromion (mean=37 cm, standard deviation=2.9 cm)

**Job characteristics**

17. Job title (secretary=8%/other supporting employee=19%/other=73%)
18. Working on a temporary contract (yes=41%/no=59%)
19. 1Number of working days per week (mean=4 days, standard deviation=1 day)
20. Number of working hours in contract per week (mean=32 hours, standard deviation=8 hours)
21. Supervising people (yes=10%/no=90%)
22. Working with hands above shoulder height during work (often=10%/seldom or never=90%)
23. Lifting or carrying >5kg at work(often=2%/once in a while=14%/seldom or never=84%)
24. Firmly squeezing with hands at work (often=8%/seldom or never=92%)
25. Repetitive tasks at work excluding computer use (seldom or never=81%/once in a while=11%/often=8%)
26. Precision mouse work (hardly ever=76%/0-1 hours per day=17%/1-2 hours per day=4%/2-4 hours per day=3%/>4 hours/day=0%)
27. Frequency of using computer and telephone at the same time at work (never=55%/sometimes=38%/often=7%/always=0%)
28. Increase in daily computer use during past year (yes=32%/no=68%)

**Computer work behavior**

29. Use of more than one computer at the same time during computer work (no=79%/sometimes=13%/regularly=4%/often=4%)
30. Total computer use hours per day at work (hardly ever=0%/0-1 hours per day=0%/1-2 hours per day=0%/2-4 hours per day=9%/4-6 hours per day=37%/6-8 hours per day=53%/>8 hours per day=1%)
31. Total computer use hours per day while working at home (never=28%/hardly ever=7%/0-1 hours per day=9%/1-2 hours per day=11%/2-4 hours per day=7%/4-6 hours per day=18%/6-8 hours per day=15%/>8 hours per day=4%)
32. Mouse use hours per day at work (hardly ever=1%/0-1 hour per day=9%/1-2 hours per day=24%/2-4 hours per day=40%/4-6 hours per day27%/6-8 hours per day=0%/>8 hours per day=0%)
33. Mouse use hours per day while working at home (never=28%/hardly ever=14%/0-1 hour per day=12%/1-2 hours per day=15%/2-4 hours per day=12%/4-6 hours per day=9%/6-8 hours per day=8%/>8 hours per day=1%)
34. Use of break and reminder software (yes=6%/no=94%)
35. Performs stretch exercises during computer work (never=69%/sometimes, often, or always=31%)
36. Often works for >1 hour without 5 min break (yes=62%/no=38%)
37. Frequency of short (<5 min) breaks during computer use (hardly ever=17%/once in a while=18%/sometimes=31%/regularly=34%)
38. Forward chin movement while looking at the monitor (yes=86%/no=14%)
39. Supports elbow, wrist, or forearm during keyboard use (yes=90%/no=10%)
40. Supports elbow, wrist, or forearm during mouse use (yes=96%/no=4%)
41. Able to touch type (yes=37%/no, look at keyboard=13%/no, look at screen and keyboard=50%)
42. Number of fingers used for typing (1-2=16%/3-9=47%/10=37%)
43. Mouse handedness (right=89%/left=3%/both=8%)
44. Mouse motor control strategy (hand only=46%/lower arm only=22%/hand and arm=31%/no movement required=1%)
45. Sitting posture (a little bent forward=32%/straight up with back on chair=29%/straight up without back on chair=14%/bent back=6%/variable=19%)
46. Measured percentage keyboard use (mean=22%, standard deviation=11%)
47. Measured percentage mouse use (mean=42%, standard deviation=11%)
48. Measured percentage idle time (mean=37%, standard deviation=9%)

**Psychosocial factors**

49. Number of overtime hours per week (mean=4.4 hours per week, standard deviation=6.5 hours per week)
50. Work continuation during formal breaks (yes=49%/no=51%)
51. Task variation (*5 question scale, range 0–12,* mean=8, standard deviation=2)
52. Effort (*Siegrist et al. 2004* [33] *, 5 question scale, range 0–20*, mean=6, standard deviation=3)
53. Reward (*Siegrist 2004* [33] *, 11 question scale, range 0–20*, mean=8, standard deviation=2)
54. Decision authority (*Karasek 1998* [32] *, 3 question scale, range 0–9,* mean=7, standard deviation=2)
55. Perceived stress (*Cohen et al. 1983* [35] *, 4 question scale, range 0–12,* mean=5, standard deviation=2)
56. Need for recovery (*Veldhoven and Broersen 2003* [36] *, Sluiter et al. 1999* [37] *, 12 question scale, range 0–12,* mean=4, standard deviation=3)
57. Number of deadlines in past 3 months (0=16%/1=14%/1-3=36%/>3=34%)
58. Current job satisfaction (never=2%/sometimes=10%/often=63%/always=25%)
59. Job satisfaction over the past 3 months (never=1%/sometimes=19%/often=64%/always=16%)
60. Increased time pressure in the last 3 months (no=50%/yes for a short time=11%/yes for a longer time=39%)
61. Burdened by increased time pressure in the last 3 months (no=56%/moderately=27%/rather=16%/very=1%)
62. Experience of stress at work (not=16%/a little=74%/quite=9%/very=0%)
63. Burdened by experience of stress at work (not=83%/a little=13%/quite=2%/very=2%)
64. Today's stress compared to normal stress (less=43%/normal=55%/more=2%)
65. Perceived tension (never=15%/sometimes=55%/few times per week=16%/>1 time per day=14%)

**Workstation setup**

66. Use of laptop for office computer work (no=88%/<desktop use=7%/equal to desktop use=2%/>desktop use=1%/always=2%)
67. Lack of space on desk for proper mouse use (never=60%/sometimes=35%/often=5%/always=0%)
68. Mouse functioning (never=76%/sometimes, often, or always=24%)
69. Monitor location relative to computer (in front=92%/left or right=8%)
70. Monitor height relative to eyes (eye level or lower=88%/higher=12%)
71. Keyboard height relative to elbows (above=15%/level to=80%/other=5%)
72. Chair height (knees higher than hips=0%/knees level to hips=94%/cannot put feet on floor=6%)
73. Keyboard >10 cm from table edge (yes=83%/no=17%)
74. Keyboard supports unfolded (yes=61%/no=39%)
75. Mouse type (standard=90%/alternative=10%)
76. Mouse location relative to keyboard (right beside=24%/further away from=37%/next to and behind=22%/in front of and next to=17%/directly in front of=0%/another place=0%)
77. Measured key activation force (mean=0.36 N, standard deviation=0.36 N)
78. Measured key displacement (mean=3.1 cm, standard deviation=0.2 N)
79. Measured knee height, footrest or floor to crease behind knees (mean=48 cm, standard deviation=3 cm)
80. Measured chair height, footrest or floor to chair seat (mean=50 cm, standard deviation=3 cm)
81. Measured monitor distance, monitor screen to nose (mean=67 cm, standard deviation=9 cm)
82. Measured elbow height, footrest or floor to elbow (mean=75 cm, standard deviation=4 cm)
83. Measured eye height, elbow to eye (mean=50 cm, standard deviation=4 cm)
84. Measured keyboard height, footrest or floor to keyboard (mean=77 cm, standard deviation=3 cm)
85. Measured keyboard distance, edge of table to keyboard (mean=24 cm, standard deviation=9 cm)
86. Measured mouse height, footrest or floor to mouse (mean=83 cm, standard deviation=65 cm)
87. Measured mouse distance, participant midline to mouse (mean=43 cm, standard deviation=7 cm)
88. Measured mouse direction, angle from participant midline to mouse (mean=53 cm, standard deviation=11 cm)
89. Measured monitor height, footrest or floor to monitor (mean=121 cm, standard deviation=10 cm)
90. Measured seat depth, front edge of chair to backrest (mean=46 cm, standard deviation=3 cm)
91. Measured monitor screen diagonal length (mean=47 cm, standard deviation=5 cm)
92. Measured keyboard tilt angle (mean=7 degrees, standard deviation=4 degrees)

**Health and pain**

93. Know acquaintances experiencing disabling symptoms (yes=29%/no=71%)
94. General health (good=82%/pretty good=15%/moderate=3%/bad=0%)
95. Neck-shoulder symptoms (no=25%/once in a while=50%/frequently or for a longer time=25%)
96. Arm-wrist-hand symptoms (no=60%/once in a while=30%/frequently or for a longer time=10%)
97. Back symptoms (no=50%/once in a while=35%/frequently or for a longer time=15%)
98. Symptoms in the past year causing disability or medical consumption (no=73%/yes in last 3 months=9%/yes but not in last 3 months=9%/yes=9%)

**Leisure time activities**

99. Number of days per week with at least 30 minutes moderate physical activity (mean=4 days, standard deviation=2 days)
100. Time in past 3 months performing strenuous physical activity (never=13%/<1 per month=12%/1-3 times per month=13%/1 per week=18%/2 per week=22%/3+ times per week=22%)
101. Strength training of upper body in last 3 months (yes=25%/no=75%)
102. Playing sports involving upper extremities (e.g. racket sports, volleyball) in last 3 months (yes=15%/no=85%)
103. Hand intensive activities during leisure time in last 3 months (yes=27%/no=73%)
104. Duration of computer use during leisure time in last 3 months (almost never=0%/0 to 1 hours per day=9%/1 to 2 hours per day=43%/2 to 4 hours per day=34%/4 to 6 hours per day=12%/6 to 8 hours per day=2%/>8 hours per day=0%)

## Authors’ original submitted files for images

Below are the links to the authors’ original submitted files for images. Authors’ original file for figure 1

## References

[1] IJmker S, Huysmans MA, Blatter BM, van der Beek AJ, Van Mechelen W, Bongers PM (2007). Shoulder office workers spend fewer hours at their computer? A systematic review of the literature. Occup Environ Med.

[2] Eltayeb S, Staal JB, Kennes J, Lamberts PH, De Bie RA (2007). Prevalence of complaints of arm, neck, and shoulder among computer office workers and psychometric evaluation of a risk factor questionnaire. BMC Musculoskelet Disord.

[3] Wahlstrom J (2005). Ergonomics, musculoskeletal disorders and computer work. Occupational Medicine.

[4] Gerr F, Marcus M, Ensor C, Kleinbaum D, Cohen S, Edwards A, Gentry E, Ortiz D, Monteilh C (2002). A prospective study of computer users: I. Study Design and Incidence of Musculoskeletal Symptoms and Disorders. AJIM.

[5] Visser B, Van Dieën JH (2006). Pathophysiology of upper extremity muscle disorders. J Electromyogr Kinesiol.

[6] Gerr F, Marcus M, Monteilh CP (2004). Epidemiology of musculoskeletal disorders among computer users: lesson learned from the role of posture and keyboard use. J Electromyogr Kinesiol.

[7] Gerr F, Monteilh CP, Marcus M (2006). Keyboard use and musculoskeletal outcomes among computer users. J Occup Rehabil.

[8] Bleecker ML, Barnes SK (2012). Exposure to keyboard/mouse use = keystrokes + mouse clicks + POSTURE: a missing variable that cannot be overstated. Occup Environ Med.

[9] Winkel J, Mathiassen SE (1994). Assessment of physical work load in epidemiologic studies: concepts, issues and operational considerations. Ergonomics.

[10] Marcus M, Gerr F, Monteilh C, Ortiz DJ, Gentry E, Cohen S, Edwards A, Ensor C, Kleinbaum D (2002). A prospective study of computer users: II. Postural risk factors for musculoskeletal symptoms and disorders. Am J Ind Med.

[11] Srinivasan D, Mathiassen SE (2012). Motor variability in occupational health and performance. Clin Biomech.

[12] Li N, Yang QL, Zeng L, Zhu LL, Tao LY, Zhang H, Zhao YM (2011). Noise exposure assessment with task-based measurement in complex noise environment. Chin Med J (Engl).

[13] Methner M, Beaucham C, Crawford C, Hodson L, Geraci C (2012). Field application of the Nanoparticle Emission Assessment Technique (NEAT): task-based air monitoring during the processing of engineered nanomaterials (ENM) at four facilities. J Occup Environ Hyg.

[14] Chen JC, Chang WR, Shih TS, Chen CJ, Chang WP, Dennerlein JT, Ryan LM, Christiani DC (2004). Using exposure prediction rules for exposure assessment: an example on whole-body vibration in taxi drivers. Epidemiology.

[15] van der Beek AJ, Mathiassen SE, Windhorst J, Burdorf A (2005). An evaluation of methods assessing the physical demands of manual lifting in scaffolding. Appl Ergon.

[16] van der Beek AJ, Frings-Dresen MH (1998). Assessment of mechanical exposure in ergonomic epidemiology. Occup Environ Med.

[17] van der Beek AJ, Mathiassen SE, Burdorf A (2013). Efficient assessment of exposure to manual lifting using company data. Appl Ergon.

[18] Bruno Garza JL, Catalano PJ, Katz JN, Huysmans MA, Dennerlein JT (2012). Developing a framework for predicting upper extremity muscle activities, postures, velocities, and accelerations during computer use: the effect of keyboard use, mouse use, and individual factors on physical exposures. J Occup Environ Hyg.

[19] Johnson PW, Hagberg M, Hjelm WE, Rempel D (2000). Measuring and characterizing force exposures during computer mouse use. Scand J Work Environ Health.

[20] Asundi K, Johnson PW, Dennerlein JT (2012). Variance in direct exposure measures of typing force and wrist kinematics across hours and days among office computer workers. Ergonomics.

[21] Bruno Garza JL, Eijckelhof BH, Johnson PW, Raina SM, Rynell PW, Huysmans MA, Van Dieën JH, van der Beek AJ, Blatter BM, Dennerlein JT (2012). Observed differences in upper extremity forces, muscle efforts, postures, velocities and accelerations across computer activities in a field study of office workers. Ergonomics.

[22] Bruno Garza JL, Eijckelhof BHW, Johnson PW, Catalano P, Katz JN, Huysmans MA, Van Dieen JH, van der Beek AJ, Blatter BM, Dennerlein JT (2013). The effect of reward and over-commitment on trapezius muscle effort and postures of the head, neck, and torso. Am J Indust Med.

[23] Eijckelhof BH, Bruno Garza JL, Huysmans MA, Blatter BM, Johnson PW, Van Dieen JH, van der Beek AJ, Dennerlein JT (2013). The effect of overcommitment and reward on muscle activity, posture, and forces in the arm-wrist-hand region – a field study among computer workers. Scand J Work Environ Health.

[24] Jensen C, Vasseljen O, Westgaard RH (1993). The influence of electrode position on bipolar surface electromyogram recordings of the upper trapezius muscle. Eur J Appl Physiol.

[25] Svendsen SW, Mathiassen SE, Bonde JP (2005). Task based exposure assessment in ergonomic epidemiology: a study of upper arm elevation in the jobs of machinists, car mechanics, and house painters. Occup Environ Med.

[26] Teschke K, Trask C, Johnson P, Chow Y, Village J, Koehoorn M (2009). Measuring posture for epidemiology: Comparing inclinometry, observations and self-reports. Ergonomics.

[27] Bruno JL, Li Z, Trudeau M, Raina S, Dennerlein JT (2012). A video-based postural assessment system to measure rotation of the shoulder during computer use. J Appl Biomech.

[28] Huysmans MA, Ijmker S, Blatter BM, Knol DL, Van Mechelen W, Bongers PM, van der Beek AJ (2012). The relative contribution of work exposure, leisure time exposure, and individual characteristics in the onset of arm-wrist-hand and neck-shoulder symptoms among office workers. Int Arch Occup Environ Health.

[29] IJmker S, Huysmans MA, van der Beek AJ, Knol DL, Van Mechelen W, Bongers PM, Blatter BM (2011). Software-recorded and self-reported duration of computer use in relation to the onset of severe arm-wrist-hand pain and neck-shoulder pain. Occup Environ Med.

[30] van den Heuvel SG, IJmker S, Blatter BM, De Korte EM (2007). Loss of productivity due to neck/shoulder symptoms and hand/arm symptoms: results from the PROMO-study. J Occup Rehabil.

[31] IJmker S, Mikkers J, Blatter BM, Van der Beek AJ, Van Mechelen W, Bonger PM (2008). Test-retest reliability and concurrent validity of a web-based questionnaire measuring workstation and individual correlates of work postures during computer work. Appl Ergon.

[32] Karasek R, Brisson C, Kawakami N, Houtman I, Bongers P, Amick B (1998). The Job Content Questionnaire (JCQ): an instrument for internationally comparative assessments of psychosocial job characteristics. J Occup Health Psychol.

[33] Siegrist J, Starke D, Chandola T, Godin I, Marmot M, Niedhammer I, Peter R (2004). The measurement of effort-reward imbalance at work: European comparisons. Soc Sci Med.

[34] Schreurs PJG, Van de Willige G, Brosschot JF, Tellegen B, Graus GMH (1993). De Utrechtse Coping Lijst: UCL. Omgaan met problemen en gebeurtenissen. Swets en Zeitlinger b.v. Lisse.

[35] Cohen S, Kamarck T, Mermelstein R (1983). A global measure of perceived stress. J Health Soc Behav.

[36] Van Veldhoven M, Broersen S (2003). Measurement quality and validity of the "need for recovery scale". Occup Environ Med.

[37] Sluiter JK, van der Beek AJ, Frings-Dresen MH (1999). The influence of work characteristics on the need for recovery and experienced health: a study on coach drivers. Ergonomics.

[38] Won EJ, Johnson PW, Punnett L, Dennerlein JT (2009). Upper extremity biomechanics in computer tasks differ by gender. J Electromyogr Kinesiol.

[39] Burnham KP, Anderson DR (2004). Multimodel inference: understanding AIC and BIC in Model Selection. Sociol Methods Res.

[40] Efron B, Gong G (1983). A leisurely look at the bootstrap, the jackknife, and cross-validation. Am Stat.

[41] Mathiassen SE, Nordander C, Svendsen SW, Wellman HM, Dempsey PG (2005). Task-based estimation of mechanical job exposure in occupational groups. Scand J Work Environ Health.

[42] Madeleine P (2010). On functional motor adaptations: from the quantification of motor strategies to the prevention of musculoskeletal disorders in the neck-shoulder region. Acta Physiol (Oxf).

[43] Mark LS, Nemeth K, Gardner D, Dainoff MJ, Paasche J, Duffy M, Grandt K (1997). Postural dynamics and the preferred critical boundary for visually guided reaching. J Exp Psychol Hum Percept Perform.

[44] Gerr F, Marcus M, Ortiz D, White B, Jones W, Cohen S, Gentry E, Edwards A, Bauer E (2000). Computer users' postures and associations with workstation characteristics. AIHAJ.

[45] Xu X, Chang CC, Lu ML (2012). Two linear regression models predicting cumulative dynamic L5/S1 joint moment during a range of lifting tasks based on static postures. Ergonomics.

[46] Starr SJ, Shute S, Thompson CR (1985). Relating posture to discomfort in VDT Use. J Occup Med.

[47] Barbe MF, Barr AE (2006). Inflammation and the pathophysiology of work-related musculoskeletal disorders. Brain Behav Immun.

[48] Samani A, Mathiassen SE, Madeleine P (2013). Cluster-based exposure variation analysis.

[49] Chang CH, Johnson PW, Dennerlein JT (2008). A wide range of activity duration cutoffs provided unbiased estimates of exposure to computer use. J Occup Environ Hyg.

[CR50] The pre-publication history for this paper can be accessed here:http://www.biomedcentral.com/1471-2474/15/292/prepub

