# Supplementary figures and images for: Prediction of trapezius muscle activity and shoulder, head, neck, and torso postures during computer use: results of a field study
Source: BMC Musculoskelet Disord. 2014 Sep 3;15:292. doi: 10.1186/1471-2474-15-292 (PMC4161866; doi:10.1186/1471-2474-15-292)

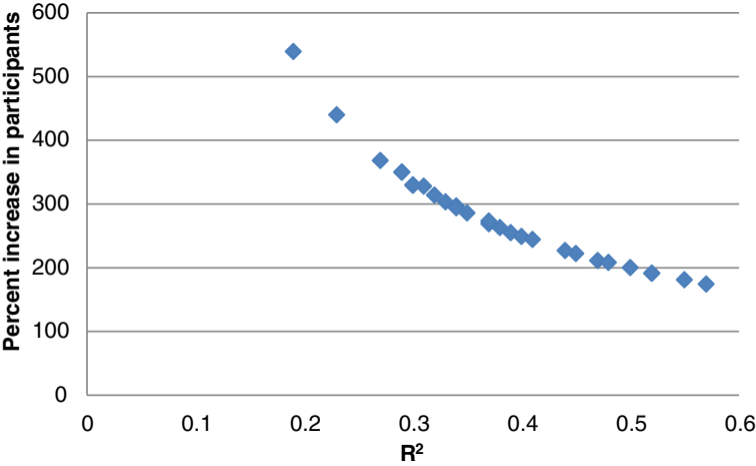

Supplement: Supplementary file 1 — Authors’ original file for figure 1 [file 12891_2013_2234_MOESM1_ESM.pdf]
